# Electro-Enhanced Gas Fermentation for Bioproduction of Volatile Fatty Acids and Alcohols

**DOI:** 10.3390/microorganisms13020249

**Published:** 2025-01-23

**Authors:** Clemens Hiebl, Werner Fuchs

**Affiliations:** Department IFA-Tulln, Institute of Environmental Biotechnology, BOKU University, Konrad Lorenz Strasse 20, 3430 Tulln, Austria; clemens.hiebl@boku.ac.at

**Keywords:** electro-fermentation, gas fermentation, sub-stoichiometric electron supply, chain elongation, *C. carboxidivorans*, *A. bacchi*

## Abstract

This study investigates sub-stoichiometric electron supply, also termed electro-fermentation, to influence product formation in gas fermentation. Two species, *Clostridium carboxidivorans* and *Alkalibaculum bacchi*, as well as a co-culture of *A. bacchi* and *Clostridium kluyveri*, were tested in batch cultures with and without an external cell potential of 800 mV. The supplied gas mixture was 50:40:10 N_2_:H_2_:CO_2_. The test unit was a single-chamber reactor with a cathode made from an electrically conducting composite of PP and black carbon. The observed current densities were generally very low, around 0.22 mA/m^2^. Despite that, a significant and reproducible change in product patterns and formation rates occurred. *C. carboxidivorans* increased the formation of acetate (+32%), butyrate (+300% relative to the control), and caproate (+600% relative to the control). In a similar manner, *A. bacchi* produced more acetate (+38%), butyrate (13 times more than the control), and caproate (only observed in the electrified setup). Additional trials using a modified gas phase composition, 80:20 H_2_:CO_2_, confirmed the finding that the application of an electric potential enhances chain elongation as well as alcohol formation. Moreover, an experiment with reversed electric polarity showed that a high cathode surface area is essential for inducing metabolic modifications. The results demonstrate that electro-fermentation holds significant potential for improving bioconversion processes aimed at producing green chemicals.

## 1. Introduction

Electroactive microorganisms (EAMs) are microbes that are capable of biochemical interaction with polarized electrodes submerged into the media [1,2]. EAM own electron transfer pathways that connect intracellular redox reactions to extracellular materials by transferring electrons across the cell membranes and walls [1,2]. In microbial electrosynthesis, an electric current is used to drive an otherwise less favorable bioreaction and thereby modify the product spectrum. A specific case is electro-fermentation. This technique is a relatively new tool to control the metabolism by applying a low electric potential [3]. According to current understanding, the applied electric potential changes the extracellular ORP (oxidizing/reducing redox potential) which can subsequently affect the intracellular ORP through the reduced/oxidized NAD (NADH/NAD^+^) balance. Intracellular ORP, representing the redox state inside a cell, can be estimated from the NADH/NAD^+^ ratio because of intracellular redox homeostasis. It is known to control gene expression and enzyme synthesis, thereby impacting the entire metabolic process and modifying metabolic pathways [4]. In contrast to other electro-biosynthesis applications, electro-fermentation involves low current instead of high current densities since the number of transferred electrons is not directly proportional to the amount of product [5]. Even though the mechanisms of the pathways and the influence of the low potential applied are still insufficiently understood, it is considered a highly promising technique in advanced biotechnological processes [6]. This study aims to study electro-fermentation for its application in gas fermentation. Gas fermentation is part of a “waste to-X” concept which aims at the conversion of low-value residues into chemicals or fuels. In an initial gasification step, organic waste materials are pyrolyzed to gaseous components and, subsequently, de novo biosynthesis from C1 compounds follows [7]. The microbial species involved in gas fermentation use H_2_ as an electron donor and CO_2_ or CO as electron acceptors to form acetate or ethanol through the so-called Wood–Ljungdahl pathway [8]. From these precursors chemicals of higher molecular weight may be subsequently produced through another microbial conversion process termed chain elongation. While gas fermentation has reached a significant level of technical maturity, the major drawback is the still limited product spectrum. Methane production and ethanol formation are currently the only processes established at the full scale [9]. Significant scientific effort utilizing advanced biotech tools such as synthetic biology or metabolic engineering is currently being made to further develop this technology [10]. Electro-fermentation also has the potential to broaden the product range as well as to substantially improve concentration levels and productivity rates [11,12]. In the present study, we provide results showing the impact of a low cell potential (800 mV) on the formation of medium-chained organic acids or their respective alcohols from gaseous substrates in pure and co-cultures.

Three sets of experiments were conducted, each consisting of an electrified triple approach and the corresponding control experiments. Two of them were investigations on pure cultures of *Clostridium carboxidivorans* and *Alkalibaculum bacchi*, respectively; the third experiment involved a co-culture of *A. bacchi* and *Clostridium kluyveri*. So far, electro-biosynthesis utilizing *C. carboxidivorans* has only been studied in a mixotrophic system employing organic carbon sources, but not in plain gas fermentation [13]. Regarding *A. bacchi*, to the best of our knowledge, no publications report on electro-fermentation utilizing this microorganism. The third bacterium involved in this study, *Clostridium kluyveri*, is known to be electroactive [14]. It has also been reported that *C. kluyveri* enhances caproate production by synergistically cooperating with acetogens in mixed microbial communities of electro-fermentation systems [15].

## 2. Materials and Method

### 2.1. Microorganisms

*C. carboxidivorans* (DSMZ 15243) is a Gram-positive, anaerobic, motile, and spore-forming bacteria. It converts CO_2_, CO, and H_2_ into acetate and is able to elongate the carbon chain up to caproic acid and also to form the respective alcohols [13,16]. Its temperature growth range lies between 24 °C and 42 °C and has its optimum at 39 °C [17]. Since the culture is capable of autotrophically producing hexanol, it has been the focus of interest in gas fermentation over the last years [16,18,19,20]

*A. bacchi* (DSMZ 22112) is a motile, Gram-negative, non-spore-forming, rod-shaped, autotrophic, and strictly anaerobic strain with the main products being ethanol and acetate. *A. bacchi* functional temperature range lies between 15 and 40 °C and has its optimum at 37 °C. The growth range extends from pH value 6.5 to up to 10.5, and has its optimum between pH 8.0 and 8.5 [21]. It is able to utilize glucose, fructose, methanol, and n-butanol as substrates, as well as H_2_/CO_2_ and CO/CO_2_.

*C. kluyveri* (DSMZ 555) is an anaerobic, Gram-positive, rod-shaped, motile, endospore-forming bacteria. It is well studied for its property to form chains up to caproic acid from ethanol and acetate alone. Its pH range lies between 6 and 7.5; it is able to grow between 19 °C and 37 °C with a growth optimum of 34 °C [22].

### 2.2. Media and Additives

Media composition is listed in Table 1, Table 2 and Table 3.

### 2.3. Experimental Procedure

Experiments were set up as triplets using serum flasks with a butyl rubber stopper and an aluminum crimp. The total volume of the flasks was 100 mL with an initial liquid volume of 30 mL leaving a headspace of 70 mL. The media served GueAd minimal media (Table 1, Table 2 and Table 3). Besides an N, P, and K source, it contained trace elements and vitamins. Precultures were prepared by a two-step enrichment procedure. N_2_ was used as inert gas component to avoid a strong pressure drop. Moreover, H_2_ was dosed in excess, on one hand to compensate for the low H_2_ solubility and to support the formation of reduced metabolites, such as medium-chain VFAs and alcohols.

**Figure 1:**
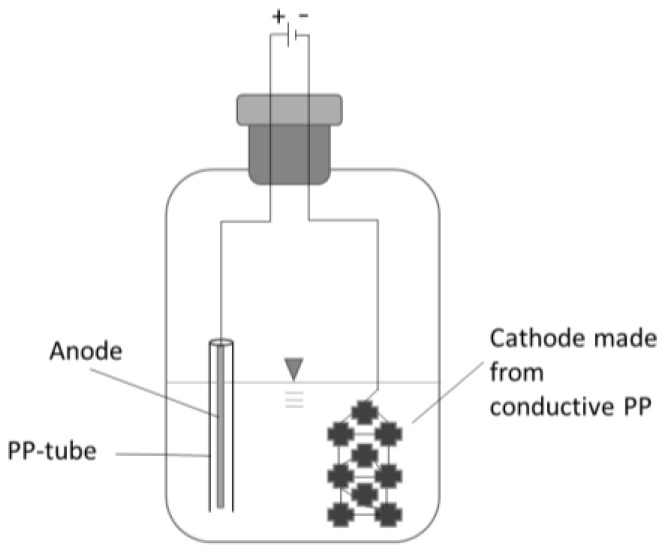
Scheme of electro flask setup.

The first cultivation was conducted according to the conditions suggested by DSMZ employing an organic substrate. Subsequently, the cultures were transferred to a similar minimal media and gas phase as used for the main experiment to avoid the carryover of unwanted complex carbon sources. Every flask setup was inoculated with 2.5 mL of the preculture free of organic substrate and the pH was set to 6.3. The flasks were sparged for 25 s with the gas mixture (N_2_:CO_2_:H_2_ = 50:10:40 *v*/*v*/*v*, N2.0 grade) to ensure a thorough exchange of gasses and the head pressure was set to 150 kPa overpressure. Every seven days the head pressure was determined; samples were taken, and the gas phase was renewed. The electrified setup contained two electrodes: the anode was a 15 cm long rod (Ø 1.6 mm) made of ER316LSI steel and loosely surrounded by a plastic cylinder to prevent unwanted short circuits (Figure 1). The cathode and carrier material for bacterial aggregation was made of nine carriers made of conducting polypropylene (PP) (a co-extrudate with 60% carbon black). The carriers were linked by a V2A wire (900 mm long, Ø 0.35 mm). The selection of cathode material was guided by key criteria: chemical and biological stability, as well as the ability to provide sufficient electron transfer capacity. Polypropylene (PP) is frequently employed as an inert carrier material to support microbial immobilization [23]. Given the low electric current applied during sub-stoichiometric electron supply, moderate electrical conductivity was deemed sufficient. However, plain PP is a very effective insulator. To address this limitation, a co-extrudate with carbon black was developed in-house. Carbon materials, such as carbon fibers, carbon cloths, activated carbon, and graphite granules, have been extensively utilized in bio-electrochemical systems by other researchers [11]. Conductive polymer blends are emerging as a promising alternative due to their unique advantages, including relatively low cost, biocompatibility, and the ability to fabricate custom-shaped electrode geometries [24]. The anodes were made of stainless steel to keep the setup simple. Steel anodes are frequently employed in electro-fermentation for their relative inertness and their mechanical strength [25]. All setups were placed in a shaking incubator (115 rpm) at 30 °C. Every triplet had a non-electrified setup as control.

### 2.4. Analysis

#### 2.4.1. Potentiostat and Current Measurement Device

The supply of electric voltage and current measurement (precision ± 0.001 mA) was conducted using an in-house constructed potentiostat with automatic data recording, typically set to a 60 min interval. The applied voltage was maintained at a cell potential of 800 mV throughout all experiments. No reference electrode was implemented; however, in external experiments, the potential versus Ag/AgClsat measurements were estimated.

#### 2.4.2. HPLC

Concentrations of fatty acids and alcohols were determined by HPLC analysis (Agilent 1260 Infinity II Series HPLC System with G4212B Diode Array Detector, Agilent Technologies, Santa Clara, CA, USA) on an IC Sep ICE-Coregel ION 300 Column (Concise Separations, San Jose, CA, USA) with a mobile phase of 0.02 M H_2_SO_4_ at a flow rate of 0.325 mL/min. The column oven and detector temperature were set to 45 °C.

## 3. Results

### 3.1. C. carboxidivorans Culture

**Figure 2:**
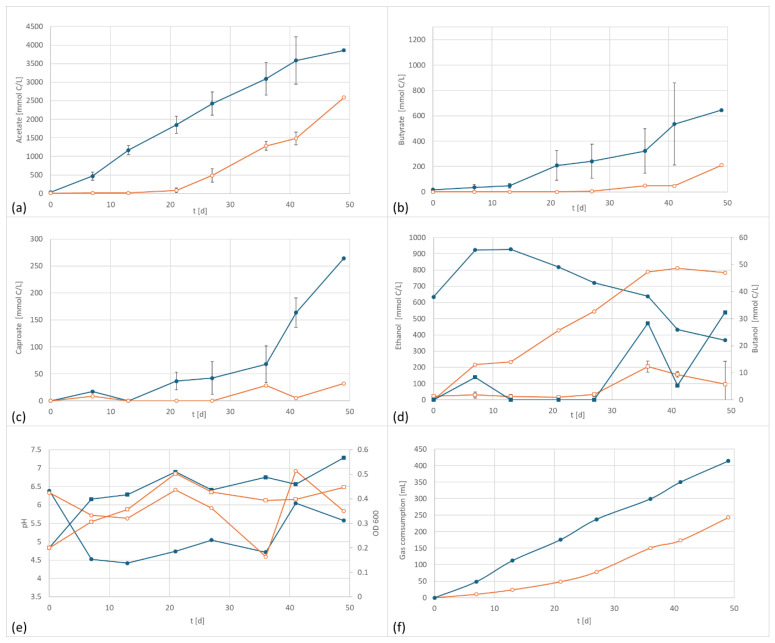
*C. carboxidivorans* product formation and performance data at 800 mV cell potential, 150 kPa 50/40/10 N_2_/H_2_/CO_2_, T = 30 °C; EF in dark blue, controls in orange; (**a**) acetate formation, (**b**) butyrate formation, (**c**) caproate formation, (**d**) formation of ethanol (circles) and butanol (squares), (**e**) pH (circles) and OD (squares), and (**f**) gas consumption; error bars represent the standard deviation of the triplicates.

In the first test series, *C. carboxidivorans* was studied. According to Cheng et al. (2022), this species shows no indication of direct extracellular electron transfer (EET) [26]. However, they also report higher alcohol production and carbon conversion efficiency at an applied electric cathodic potential of −757 mV. Results obtained in the current experiments are depicted in Figure 2a–f.

As shown in Figure 2, the application of a potential of +800 mV resulted in a significant and reproducible shift in product formation and pattern. The formation of acetate started earlier than the control and resulted in higher final concentrations. Butyrate and caproate formation were also affected by the applied potential. The butyrate concentration was three times higher and the caproic content was eight times as high as in the control setup. In contrast to other studies, the ethanol formation was negatively affected. However, since ethanol serves as an electron donor for chain elongation, additionally produced ethanol is very likely consumed for the synthesis of products of longer chain length.

The electrified setup produced 3850 mg/L acetate, 645 mg/L butyrate, and 182 mg/L caproate, versus 2590 mg/L acetate, 211 mg/L butyrate (32% of electrified) and 32 mg/L caproate.

### 3.2. A. bacchi Culture

In a second test series, a similar setup was used for experiments with *A. bacchi*. This strain has a range of growth between pH 6.5 and pH 10.5 [21]. The relatively high pH range is rather unusual for an acetogenic strain. However, it makes this species a promising candidate for products with higher chain length since low pH values are reported to be in conflict with efficient chain elongation [27,28,29]. In the current experiments, the starting pH value of the media was 6.3, the same value as for all other trials and slightly lower than the optimal range of *A. bacchi*.

**Figure 3:**
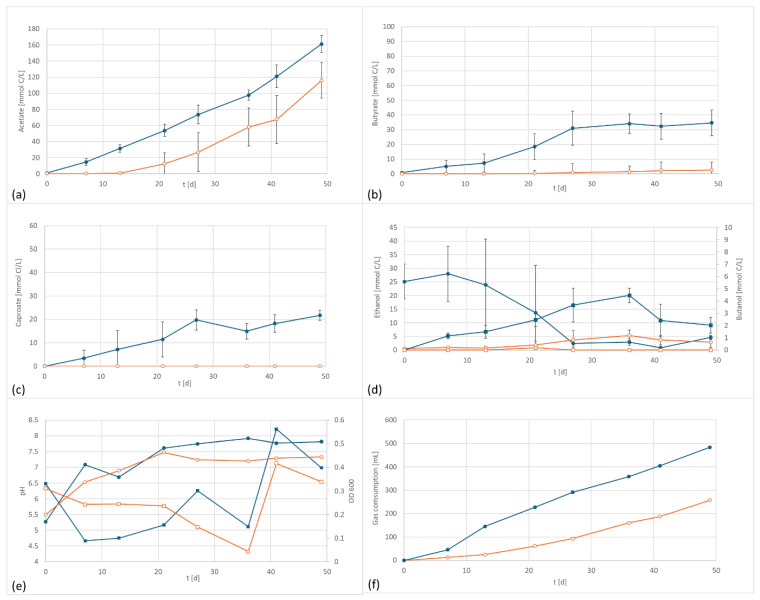
*A. bacchi* product formation and performance data at 800 mV cell potential, 150 kPa 50/40/10 N_2_/H_2_/CO_2_, T = 30 °C; EF in dark blue, controls in orange; (**a**) acetate formation, (**b**) butyrate formation; (**c**) caproate formation, (**d**) formation of ethanol (circles) and butanol (squares), (**e**) pH (circles) and OD (squares), and (**f**) gas consumption; In the control the ethanol is continuously increasing whereas the electrified flasks reached a peak concentration after the amount of ethanol starts to decline again. This is an indicator for enhanced ethanol consumption which can be an indicator for chain elongation, also shown by the formation of medium chained components.

Similar to the first experiment, accelerated acetate and butyrate formation was observed under the application of an electric potential (Figure 3). The acetate values were about 30% higher, and butyrate reached 15 times the amount of butyrate of the control. Also, caproate was discovered in the electrified setup whereas it was not found in the control. The final level of caproate in this setup was even higher than in the setup with the well-known caproate producer *C. carboxidivorans*. The electrified setup produced 3631 mg/L acetate, 712 mg/L butyrate and 353 mg/L caproate. In comparison, the control produced 2026 mg/L acetate (55% electrified), 48 mg/L butyrate (6% electrified), and 0 mg/L caproate. The formation of caproate by *A. bacchi* has not been described in the literature so far and it only occurred when a potential was applied. Again, the findings were supported by the total gas consumption, which was 483 mL in the electrified setup, almost twice the amount consumed by the control, 258 mL.

### 3.3. Co-Culture

**Figure 4:**
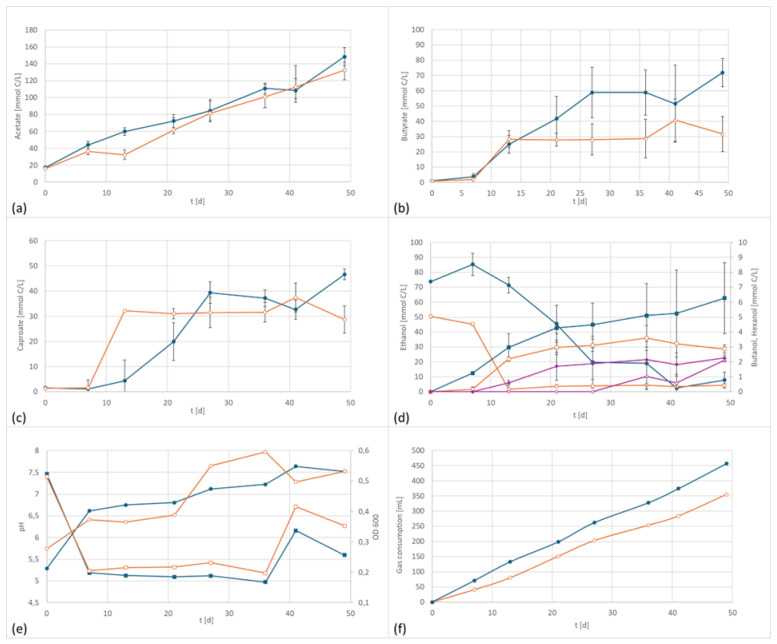
Co-culture product formation and performance data at 800 mV cell potential, 150 kPa 50/40/10 N_2_/H_2_/CO_2_, T = 30 °C; EF in dark blue, controls in orange; (**a**) acetate formation, (**b**) butyrate formation; (**c**) caproate formation, (**d**) formation of ethanol (circles) and butanol (squares), purple line: the concentration of hexanol over time, (**e**) pH (circles) and OD (squares), and (**f**) gas consumption.

In the final series co-cultivation of two species, a mixed culture of the alkaliphilic acetogenic strain *A. bacchi* and the chain elongating clostridia *C. kluyveri*, was tested. *C. kluyveri* does not grow autotrophically but is one of the most studied strains using reverse beta-oxidation for chain elongation [22,27,28,29]. Moreover, it is able to reduce the formed organic acids into their respective alcohols. Chain elongation is favored by higher pH values, whereas the formation of alcohol requires low pH values [27]. It was, therefore, presumed that cultivation of an acetogenic strain at a more basic pH level should lead to enhanced medium chained fatty acids (MCFA) formation of higher chain length. However, in these tests, the differences in product spectrum between the electrified setup and the control are not as pronounced as in the pure culture investigations, e.g., the graphs of the acetate concentration were almost parallel (the small acetate content at day 0 derived from the inoculum of *C. kluyveri* which was pre-cultivated on an acetate/ethanol mixture). The biggest difference between the two setups is the formation of about 50% more butyrate in the electrified setup. Regarding other metabolites, the picture was more complex: Caproate was produced in relatively similar quantities, and higher carboxylic acids were not observed. Temporarily higher ethanol concentrations were observed in the electrified setup but were later re-consumed. Butanol levels were slightly higher whereas hexanol formation was delayed but ultimately reached a similar concentration (Figure 4).

The electrified setup had a final concentration of 4459 mg/L acetate, 1583 mg/L butyrate, 902 mg/L caproate, 116 mg/L n-butanol as well as 116 mg/L ethanol at the end of the fermentation. For comparison, the control had the following concentrations at the end of the incubation period: 3984 mg/L acetate (90% electrified), 698 mg/L butyrate (44% electrified), 556 mg/L caproate (60% electrified), 68 mg/L butanol (56% of electrified), and 117 mg/L ethanol (1:1 to electrified). Similar to the pure cultures, more gas was consumed in the electrified setup. However, the only product concentration that differed significantly from the control was butyrate.

An overview of all final concentrations and the average volumetric production rates over the test period calculated in C mmol/L is presented in Table 4 and Table 5.

For almost all metabolites, the standard deviations of the electrified triplicates were significantly higher than those of the controls. The exact reason for this remains speculative. We assume that the self-designed cathodes, which were evidently not entirely identical, contributed to the observed variance. Additionally, pre-cultures were cultivated under non-electrified conditions, and the adaptation of microorganisms to the modified environment may have amplified certain inhomogeneities in the starting conditions.

### 3.4. Electric Current Flow

The electric current observed in our experiments was very low, in the range of 2 to 6 µA. It remained at the same level throughout all experiments and no obvious trend, neither up- nor downwards was observed. The carriers were self-constructed, and the exact surface area of the cathode is not known; however, we estimate it to be ~186 cm^2^. Consequently, the current density lies at 0.22 mA/m^2^: Typical ranges of current densities in bio-electrosynthesis reported in the literature are between 2 A/m^2^ [30] and 10.9 A/m^2^ [31] for electro-fermentation the densities lie between 462 mA/m^2^ [32] and 2.74 mA/m^2^ [33]. Lower current densities make the processes more economically attractive in terms of electricity costs and also reduce the risk of unwanted side reactions. One potential reason for the observed low current densities is the absence of any externally added electron mediator. However, to which extent the direct electron uptake or the mediators produced by the microorganisms can only be speculated.

To verify the hypothesis that the electric current acts only as a stimulant and the predominant source of redox equivalents is H_2_ from the headspace, the provided amount of ‘electric’ redox equivalents was calculated.

With the Faraday constant, representing the electric charge of one mole of elementary carriers, the moles of electrons per time can be calculated using the following formula [34]:

$$n\left(electrons\right)=\frac{I\;*\;t}{F}$$

At an electric current of 4 µA, 3.6 × 10^−6^ mol of electrons are delivered at the cathode. Four electrons (corresponding to two moles of H_2_) are required to reduce CO_2_ to acetate [35]:

CO_2_ + 4H^+^ + 4 e^−^ → ½ CH_3_COOH + H_2_O

Based on the equation above, the maximum acetate electro-formation rate is 4.48 × 10^−4^ mmol per flask per day which corresponds to a productivity of 0.90 mg/(L.d). For comparison, the acetate formation rates for the first 21 days of the tests were calculated. On average 88, 76, and 100 mg/(L·d) were observed in the electrified setups of *C. carboxidivorans*, *A. bacchi*, and the co-culture, respectively. Obviously, the measured values exceeded by far the potential acetate formation from electron supply. This confirms that the reaction works by an indirect mechanism boosting the metabolic pathways for acetate and butyrate formation. The necessary redox equivalents for CO_2_ reduction were still supplied by dissolved H_2_ gas as indicated by the term ‘sub-stoichiometric electron flow’ used for this kind of microbial electrosynthesis [5].

Generally, it was observed the impact of the electric potential on the growth rate is less pronounced than its effect on the metabolite pattern. This supports the assumed mechanism of action, that a small current consumption stimulates redox-balancing pathways [36] and that the external potential influences internal regulatory functions in response to the exterior redox potential [37]. Unfortunately, the exact means of influence still require further investigation. However, a few studies revealed further details. They have shown that the sub-stochiometric electron supply has an impact on the NADH/NAD+ [37] as well as the NADPH/NADP+ ratio [38] being the central mechanisms for the regulation of the redox balance within a cell. By means of transcriptome analysis, the later study confirmed that the electric potential up- or down-regulates the gene expression of essential metabolic enzymes. In *Zymomonas mobilis*, it led, among other modifications, to the higher expression of enzymes involved in ethanol and succinate biosynthesis. Moreover, it was demonstrated that the manipulation of essential electricity-sensing genes amplified the induced metabolic change [38].

### 3.5. Cathode Potential

**Figure 5:**
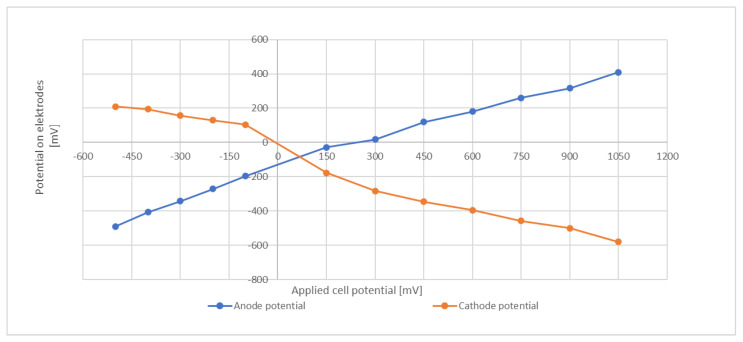
Electrode potentials at different cell voltages.

In an attempt to characterize the electrode potentials, an abiotic test that included a reference electrode was conducted. The same media and temperature as in the batch trials were employed and the media was flushed with N_2_ to remove dissolved O_2_. Several repetitions confirmed that the obtained values were well reproducible. The applied cell potential was from −500 mV (cathode and anode reversed) to +1050 mV. Results are shown in Figure 5.

At a total cell potential of +800 mV, the potential of the cathode was about −480 mV to −500 mV. This level is around the redox potential of ferredoxin [39] and is sufficiently negative to drive the NADP/NADPH redox couple toward its reduced form, e.g., in their investigations of an external potential on the heterotrophic bacterium *C. acetobutylicum* Kim and Kim (1988) proposed an electrochemical mechanism that enhances transfer of electrons to NAD(P)+ via the ferredoxin-NAD(P)+ reductase [40]. However, it must be emphasized that these abiotic tests give only an indication and do not necessarily provide the true potential within the biological system. The actual electrode potential might be subject to modification due to the microbial activity and the products formed.

### 3.6. Counterreaction

One remaining unknown is the counterreaction delivering the electrons at the anode side. In many similar bio-electrochemical systems this reaction is O_2_-formation through anodic oxidation of water. However, no indication of oxygen formation was observed, neither the redox indicator nor a luminescent oxygen sensor which was installed in the potential measurement described before showed any signs of O_2_ presence. Another option is slow dissolution of the metallic anode material, but no obvious corrosion was noted.

With a view to the redox ladder, the most likely reaction is the extraction of electrons from molecular H_2_. The so-called hydrogen oxidation reaction is the well-known anodic reaction taking place in a fuel cell [41]. It can be presumed that due to the observed low current flow only a tiny fraction of the H_2_ is oxidized while the availability of dissolved H_2_ for microbial metabolism remains largely unchanged. Nevertheless, for the current status, the final answer to the question of the anodic reaction remains open.

### 3.7. Comparison to Literature Data

Electro-fermentation has been studied by several authors. However, most studies related to the topic of medium-chained VFAs or alcohols include complex carbon sources such as yeast extracts [12], digested rice straw, or other cellulose materials [42]. Such investigations have shown the productivity under the application of a low potential improved alcohol formation in *Clostridium acetobutylicum* [43]. A low potential applied seemed to facilitate the formation of butanol directly from acetate without the need to go over the formation steps of butyrate from butyrate-CoA [44]. Other studies employed acetic acid/ethanol mixtures to specifically investigate chain elongation. Enhanced chain elongation through low electric current stimulation was reported by Ren et al. [45]. They observed a 21% increase in caproate formation after 150 days in a mixed culture where *Rhuminococcus* played the dominant role for caproate formation and lactate was acting as an electron donor.

There are only a few reports on the fermentation of gaseous substrates using bioelectrical systems. Arends et al. [46] employed a mixed microbial culture to generate isopropanol, utilizing CO_2_. In their setup, the electron flow served to provide reduction equivalents and was therefore relatively high. It was set to a fixed value of 5 A/m^2^ (total current: 50 mA). The achieved concentrations were 670 mg/L of butyrate and 820 mg/L of Isopropanol, respectively, after 70 days of fermentation.

For comparison, in the current experiments, the highest product concentrations of pure cultures were achieved with *A. bacchi* after 49 days under electrification which were: 3.600 mg/L acetate, 700 mg/L butyrate, and 350 mg/L caproate.

According to the findings of Arends and coworkers, 18.6% and 21.8% of the supplied electrons can be attributed to the reduction in C2 to butyrate and isopropanol, respectively. The actual cathode potential recorded was between −1140 mV and 1.250 mV, respectively, which is much lower than the potential presumed in the current investigations. The cathode potential in the current investigations is somehow comparable to the setup of Rovira-Alsina et al. 2021 [31], also aiming at electrosynthesis of organic compounds from CO_2_. Therein a fixed cathode potential of -600mV was applied. In this study, a starting cell potential of around 3.5 V gradually going up to 8–10 V was observed [30]. An overview of results found in the literature is presented in Table 6.

### 3.8. Verification Experiments

To verify the observations described above, the experiments were repeated two times with small variations: (i) same conditions as above but with reversed electric polarity (positively charged conductive polymer cathode); and (ii) normal polarity but applying a modified composition of the headspace gas, H_2_:CO_2_ = 80:20.

**Figure 6:**
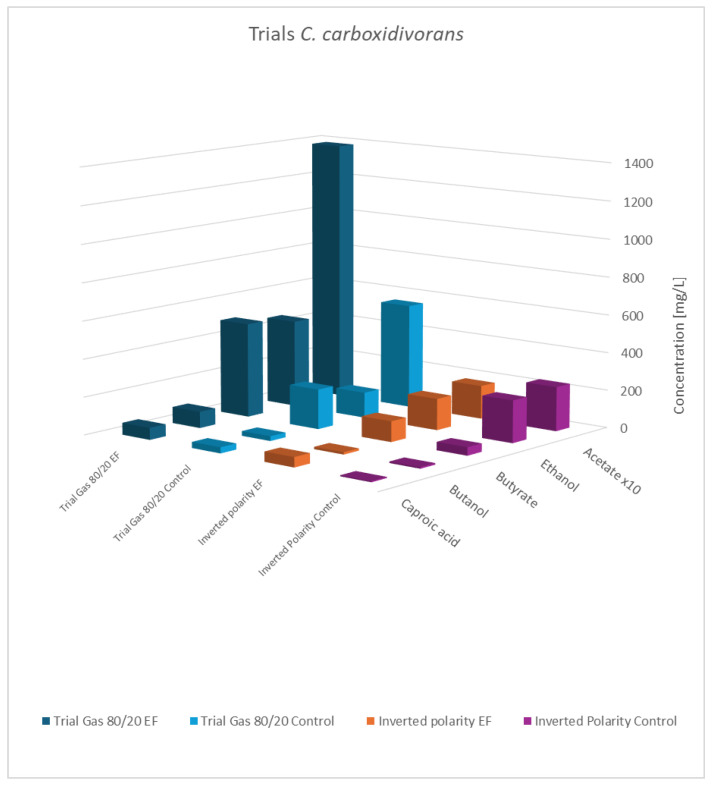
Final concentration of fatty acids/alcohols of *C. carboxidivorans*: rows 1 and 2 gas mixture 80:20 H_2_:CO_2_; normal polarity vs. control; rows 3 and 4: gas mixture 50:40:10 N_2_:H_2_:CO_2_, inverted polarity vs. control; acetate values are reduced to 1/10th of the actual value.

**Figure 7:**
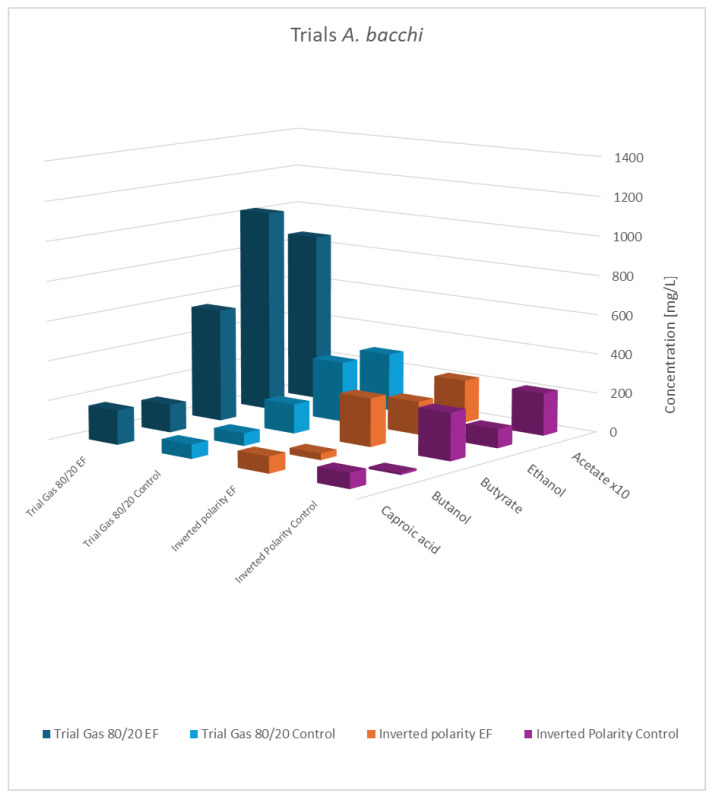
Final concentration of fatty acids/alcohols of *A. bacchi*: rows 1 and 2 gas mixture 80:20 H_2_:CO_2_; normal polarity vs. control; rows 3 and 4: gas mixture 50:40:10 N_2_:H_2_:CO_2_, inverted polarity vs. control; acetate values are reduced to 1/10th of the actual value.

An overview of the results is presented in Table 7, Figure 6 and Figure 7.

As shown, the principal results for the modified gas composition were similar to the findings obtained before. Again, the pure cultures showed the highest productivity, while the influence of a potential applied to the co-culture was still observable but less pronounced. As expected, the results with swapped polarities were completely different, and generally, no impact of electric potential was observed. This verifies that a biocathode with a high surface area and negatively charged potential is the principal pre-requisite to effectivity influence bacterial metabolism. This experiment also addresses a concern regarding the previously described experiments: the controls not only lacked an applied cell potential but also did not include any electrodes. The additional results confirmed that the presence of the electrode material alone was not responsible for the observed effects.

## 4. Conclusions

Electro-fermentation using sub-stoichiometric electron flow is an emerging tool for controlling biotechnological processes. Unlike other forms of electro-biosynthesis, it employs minimal electric currents to influence metabolic activity. This study identifies two species in which the application of low voltage in a single-cell system significantly impacts productivity and the product spectrum. The results confirm that this innovative approach can be successfully applied to gas fermentation of CO_2_ and H_2_ mixtures. At an applied cell potential of 800 mV, the electrified tests demonstrated increased gas consumption and a corresponding rise in productivity of up to 300%. The detection of metabolites previously undocumented in the literature for specific species highlights the potential of this application. The general applicability of this novel biochemical tool was verified in experiments using a modified gas composition. Moreover, the study provides clear evidence that cathode configuration plays a decisive role in process efficiency. However, significant uncertainties remain, such as those related to the electron-producing counterreaction at the anode. Further investigations are essential to deepen our understanding of the underlying biochemical and electrical mechanisms, paving the way for the full exploitation of this technique in producing green chemicals via gas fermentation.

## Figures and Tables

**Table 1:** Composition of the GueAd medium.

Species	Concentration [g/L]
KH_2_PO_4_	1.000
NaCl	1.000
NH_4_Cl	1.000
MgSO_4_·7H_2_O	0.211
K_2_CO_3_	1.000
CaCl_2_·2H_2_O	0.040
Resazurin (1% *w*/*v*)	1 mL/L
	[mL/L]
Wolin’s trace metal solution	10
Wolfe’s vitamin solution	10
Na_2_S·9 H_2_O	0.5 g/L
pH = 6.3	

**Table 2:** Composition of Wolin’s trace metal solution.

Species	Concentration [g/L]
Nitrilotriacetic acid	1.5
MgSO_4_·7H_2_O	3
MnSO_4_·H_2_O	0.5
NaCl	1
FeSO_4_·7H_2_O	0.1
CoSO_4_·7H_2_O	0.18
CaCl_2_·2H_2_O	0.1
ZnSO_4_·7H_2_O	0.18
CuSO_4_·5H_2_O	0.01
KAl(SO_4_)_2_·12H_2_O	0.02
H_3_BO_3_	0.01
Na_2_MoO_4_·2H_2_O	0.01
NiCl_2_·6H_2_O	0.03
Na_2_SeO_3_·5H_2_O	0.0003
Na_2_WO_4_·2H_2_O	0.0004

**Table 3:** Composition of Wolin’s vitamin solution.

Species	Concentration [mg/L]
Biotin	2
Folic acid	2
Pyridoxine-HCl	10
Thiamine-HCl	5
Riboflavin	5
Nicotinic acid	5
Ca-D-pantothenate	5
Vitamin B12	0.1
p-Aminobenzoic acid	5
(±)-α-Lipoic acid	5

**Table 4:** Final product composition of experimental setups of this study.

		Acetate [C mmol/L]	Butyrate [C mmol/L]	Caproate [C mmol/L]	Ethanol [C mmol/L]	n-Butanol [C mmol/L]
*C. carboxidivorans*	EF	128	29	9	16	3
	control	86	10	7	4	2
*A. bacchi*	EF	161	35	22	5	2
	control	116	3	0	3	0
Co-culture	EF	148	72	47	8	6
	control	133	32	29	8	4

**Table 5:** Average volumetric production rate values over the entire 49-day test period.

		Acetate [C mmol/(L·d)]	Ethanol [C mmol/(L·d)]	Butyrate [C mmol/(L·d)]	n-Butanol [C mmol/L·d)]	Caproate [C mmol/(L·d)]
*C. carboxidivorans*	EF	2.622	−0.576	0.598	0.035	0.279
	control	1.760	0.063	0.195	0.052	0.034
*A. bacchi*	EF	3.292	−0.647	0.708	0.124	0.444
	control	2.367	0.072	0.053	0.000	0.000
Co-culture	EF	3.029	−1.420	1.467	0.128	0.951
	control	2.708	0.075	0.646	0.075	0.586

**Table 6:** Literature values of comparable studies addressing fermentation of gaseous substrates using bioelectrical systems.

Substrate	Reactor Type	Hac [mM C]	HBu [mM C]	HCa [mM C]	EtOH [mM C]	BuOH [mM C]	Current Density [mA/m^2^]	Applied Cathode Potential [mV]	Cell Potential [V]	Reference
CO_2_	H-Cell	3.74	0.6	n.r.	1.24	-	-	−574 vs. SHE	-	[47]
CO_2_	Tilted trickle bed	34.7	87.5	-	3.4 (final conc)	5.4	2.74	−800 vs. SHE	-	[33]
N_2_/CO_2_	Serum flask	200	420	160	n.r.	n.r.	−126,000	1.02	-	[48]
H_2_/CO_2_ 80/20	Tilted trickle bed	28.98	27.85	148.26	46.88		550	−800 vs. SHE	9.82	[49]
N_2_/H_2_/CO_2_ 50/40/10	Serum flask	160	35	22	0	0	0.22	−500	0.8	*
H_2/_CO_2_ 80/20	Serum flask	216	14	4.2	25	7.6	0.25	−500	0.8	*

* In this study, the highest values achieved with *A. bacchi* are listed.; n.r. = not reported

**Table 7:** Average volumetric production rates of the verification trials.

			Acetate [C mmol/(L·d)]	Ethanol [C mmol/(L·d)]	Butyrate [C mmol/(L·d)]	n-Butanol [C mmol/L·d)]	Caproate [C mmol/(L·d)]
Reversed polarity	*C. carboxidivorans*	EF	1.052	0.141	0.093	0.013	0.056
		control	1.397	0.174	0.035	0.010	0.008
	*A. bacchi*	EF	1.499	0.126	0.202	0.031	0.077
		control	1.282	0.074	0.195	0.010	0.072
80:20 = H_2_:CO_2_	*C. carboxidivorans*	EF	9.055	0.428	0.412	0.060	0.150
		control	3.622	0.180	0.120	0.026	0.031
	*A. bacchi*	EF	5.651	0.881	0.509	0.149	0.175
		control	1.923	0.261	0.133	0.068	0.072

## Data Availability

The raw data supporting the conclusions of this article will be made available by the authors upon request.
